# Presence of BAG3 protein in serum samples from patients affected by psoriasis

**DOI:** 10.1186/s12967-024-05271-y

**Published:** 2024-05-15

**Authors:** Antonia Falco, Anna Basile, Annunziata Raimondo, Giulia Guglielmi, Alessandra Rosati, Margot De Marco, Maria Caterina Turco, Maria Pascale, Serena Lembo

**Affiliations:** 1 Department of Medicine, Surgery and Dentistry, Schola Medica Salernitana, University of Salerno, Baronissi, SA Italy; 2 FIBROSYS s.r.l. Academic Spin-off, University of Salerno, Baronissi, SA Italy; 3 Department of Pharmacy, University of Salerno, Via Giovanni Paolo II, Fisciano, SA Italy

**Keywords:** BAG3, Psoriasis, Cytokines

*To the Editor*,

Due to its systemic nature, psoriasis is at risk of progression to severe forms, the pathogenetic mechanisms of which are not well understood [1]. The identification of previously unrecognized factors involved in the complex landscape of psoriasis development could be useful for monitoring the progression of the disease and the response to therapies, as well as identifying patients who are at risk to develop severe forms.

In this context, we report an observation carried out in our laboratory, that revealed the presence of a hitherto unidentified component, the BAG3 protein, in the serum of a fraction of patients affected by psoriasis.

We analyzed, using an in-house ELISA, the levels of BAG3, a protein hardly detectable in the serum of healthy subjects, in serum samples from 31 patients (70% male; mean age *±* SEM: 59.5 ± 2.6 years) affected by psoriasis. Twenty-one patients were receiving biological therapies targeting IL-17 (*N* = 10), TNF-α (*N* = 7) or IL-23 (*N* = 4), while the remaining ten were treated with immunosuppressants (*N* = 7) or a PDE4 inhibitor (*N* = 3). We identified two groups with significant (*p* < 0.0001, Mann-Whitney test) differences in BAG3 levels. Indeed, BAG3 was undetectable in sixteen of the samples, while it was clearly present in about a half (*N* = 15) of the patients, with a mean concentration of 126.0 *±* 48.0 pg/ml (mean *±* SEM) (Fig. 1A). This difference did not appear to be related (*p* = 0.501) to differences in therapy. The mean PASI scores were 2.4 *±* 0.53 SEM and 6.7 *±* 0.53 SEM for BAG3- negative and -positive patients, respectively. The difference was highly significant (*p* < 0.0001), although PASI scores were often low.

**Fig. 1:**
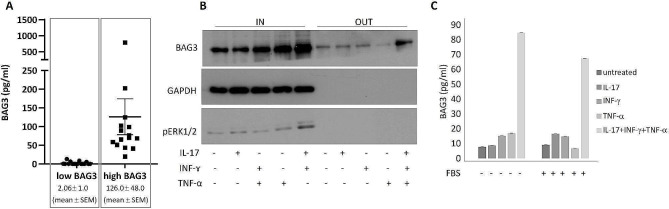
Analysis of BAG3 presence in serum samples from psoriasis patients and of its release induced by cytokines in keratinocytes. **(A)** Serum samples were analysed by a proprietary BAG3 ELISA kit. The study was approved in accordance with the ethical standards of the responsible committee on human experimentation. **(B)** Human keratinocytes HaCaT cells (CLS Cell Lines Service GmbH; Eppelheim, Germany), at 80% confluence, were treated with IL-17 A (TP723199 OriGene) [100 ng/ml], TNF-α (TP723451 OriGene) [20 ng/ml] or INF-γ (TP723162 OriGene) [20 ng/ml], alone or in combinations, in the absence or presence of 10% foetal bovine serum (FBS), for 16 h at 37 °C in a 5% CO2 atmosphere. Cell viability was not affected by treatments (data not shown). Total protein extracts from cells (IN) and proteins from supernatants (OUT) were analysed by western blotting (WB) using a proprietary anti-BAG3 pAb. An anti-pERK1/2 pAb (#9101, Cell signaling) was used to verify the cytokine-dependent cell activation. GAPDH was checked by a monoclonal antibody (sc-32,233, Santa Cruz Biotechnology) as an intracellular protein control. **(C)** BAG3 content was analysed in HaCaT supernatants by ELISA. Data were obtained from triplicate samples and confirmed in two separate experiments. Error bars indicate SD.

BAG3 is an intracellular multidomain protein expressed upon stressful conditions. By interacting with different partners, it regulates various cellular functions, including apoptosis, autophagy and motility. Its release is documented in very few pathological conditions: pancreatic cancer [2]; advanced-stage or acutely decompensated heart failure [3]; systemic sclerosis [4]. In our opinion, two features of BAG3 are interesting in relation to the study of psoriasis. First, we found that its release was induced in keratinocytes following the combined action of three cytokines: IL-17, TNF-α and IFN-γ (Fig. 1B), which are produced in the psoriatic environment [5]: this can explain the presence of BAG3 in the serum of patients affected by the disease. Furthermore, BAG3 can specifically bind monocytes/macrophages, inducing their activation and the production of various cytokines [2]; its release could therefore stimulate the inflammatory/immune response, increasing the severity of the disease.

These findings document for the first time the presence of BAG3 protein in the blood of psoriasis patients, identifying a new factor whose possible role in the disease should apparently be studied. Furthermore, the recognition of two distinct groups of patients, in only one of which the presence of BAG3 protein is detected in the serum, could pave the way for further studies, to evaluate possible connections of the presence of circulating BAG3 with specific features of the disease.

## Data Availability

Data generated during the current study are available from the corresponding author on reasonable request.
